# Cutaneous Lymphadenoma: A Case of Recurrence after Shave Excision

**DOI:** 10.1155/2021/5543404

**Published:** 2021-03-10

**Authors:** Fateme Rajabi, Kambiz Kamyab, Alireza Firooz

**Affiliations:** ^1^Center for Research & Training in Skin Diseases and Leprosy, Tehran University of Medical Sciences, Tehran, Iran; ^2^Network of Dermatology Research (NDR), Universal Scientific Education and Research Network (USERN), Tehran, Iran; ^3^Department of Dermatopathology, Razi Hospital, Tehran University of Medical Sciences, Tehran, Iran

## Abstract

Cutaneous lymphadenoma (CL) is a rare skin tumor supposedly derived from the pilosebaceous unit. Since its description in 1987, fewer than 60 cases have been documented. Herein we report a case of CL presenting as a small nodule on the forehead of a young female. The lesion recurred two years after shave excision of a similar lesion. The histopathological examination revealed interconnected islands, sheets, and trabeculae consisting of two distinct types of cells within a sclerotic stroma, a peripheral rim of palisading basophilic cells, and central epithelial cells with eosinophilic to clear cytoplasm. A dense infiltration with prominent lymphocytes and few plasma cells dominated the stroma and permeated the epithelial nests. This case represents the recurrence of this type of skin tumor after shave excision and thus highlights the importance of complete margin-free excision of such lesions.

## 1. Introduction

Cutaneous lymphadenoma (CL) is a rare adnexal tumor that usually presents as an asymptomatic skin-colored nodule on the head and neck region [1]. Though the clinical features of this tumor are fairly nonspecific with numerous clinical differential diagnoses, the histopathological findings are characteristic. It consists of well-circumscribed epithelial nests embedded in a desmoplastic stroma with heavy infiltration of lymphocytes [2]. The nests are composed of two cell lines, a peripheral rim of small basaloid cells and an aggregate of clear cells. CL is an overlooked diagnosis that is often mistaken for basal cell carcinoma, trichoblastoma, and lymphoepithelioma-like carcinoma of the skin (LELC) [3]. Herein, we describe a case of CL presenting as a skin-colored nodule on the forehead of a young female that recurred after shave excision.

## 2. Case Presentation

**Figure 1:**
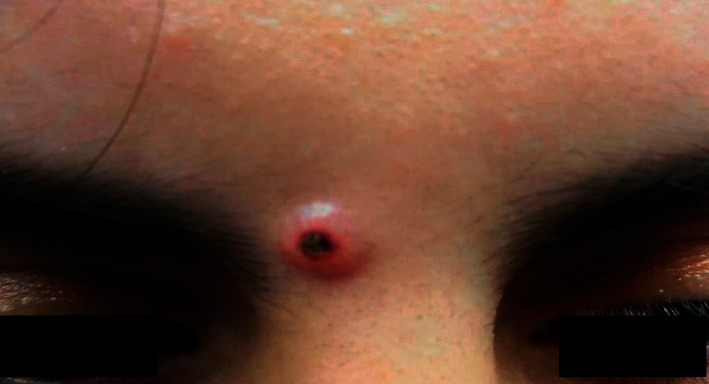
Clinical photograph of an indolent, asymptomatic nodule on the forehead.

An otherwise healthy 21-year-old woman presented for evaluation of a skin lesion on her forehead which had first appeared about seven years earlier. It had gradually grown to a 3–4 mm papule over four years when it was removed via shave excision. Approximately six months before visiting our department the lesion reemerged with a considerably faster growth rate that resulted in a 5–6 mm papule. Physical examination revealed a smooth skin-colored superficial papule with a central crust between the eyebrows (Figure 1). An excisional biopsy with a 3 mm margin was performed for histopathologic evaluation.

**Figure 2:**
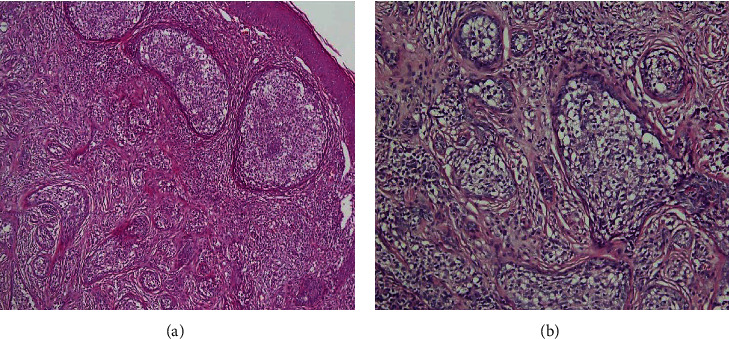
(a) Histopathological findings demonstrate ill-defined interconnected islands of epithelial cells within a sclerotic stroma (4x). (b) The epithelial cells have eosinophilic to clear cytoplasm and mild pleomorphic vesicular nuclei (10x). A peripheral rim of palisading basophilic cells surrounds the epithelial islands. A prominent lymphocytic infiltration and few plasma cells that spill over to the epithelial nests is also present (hematoxylin-eosin).

The histopathological examination revealed a dermal nodule of interconnected islands, sheets, and trabeculae of epithelial cells within a sclerotic stroma (Figure 2). The epithelial cells had eosinophilic to clear cytoplasm and mild pleomorphic vesicular nuclei. A peripheral rim of palisading basophilic cells surrounded the epithelial islands. A sclerotic stroma with dense infiltration of inflammatory cells contained the islands. The infiltration prominently consisted of lymphocytes and few plasma cells that permeated the epithelial nests. These histopathological findings were in favor of CL.

## 3. Discussion

CL is a rare skin tumor supposedly derived from the pilosebaceous unit. The tumor was first described by Santa Cruz and Barr in 1987 [4]. Since then fewer than 60 cases have been documented under different names such as cutaneous lymphoepithelial tumor, epithelial-lympho-histiocytic tumor, and adenomatoid trichoblastoma. The tumor usually affects middle-aged individuals, males more often than females, but it could affect all walks of life as even congenital lesions have been described [3, 5]. The tumor usually presents as an indolent, erythematous, or skin-colored, small nodule within the head and neck region.

The characteristic histopathology of lymphadenoma consists of a well-circumscribed but un-encapsulated nodule of epithelial nests embedded in a desmoplastic stroma both of which are heavily infiltrated by lymphocytes. The epithelial lobules can connect to the epidermis and extend down to the subcutaneous fat. The lobules are composed of two cell lines, a peripheral rim of small basaloid cells and an aggregate of large cells with faint eosinophilic cytoplasm, vesicular nuclei, and prominent nucleoli (clear cells). Ductal differentiation rimmed with central glassy cuticles, rudimentary follicular structures (papillary mesenchymal bodies), and compact keratinization are found on occasion [2]. The lymphocytic infiltration consists of mature B- and T-cells, few histiocytes (CD68+), and Langerhans cells (S100+, CD1a+) that can also form germinal center-like structures within the stroma [5, 6].

Though immunohistochemistry studies are not essential, they may help in distinguishing the differential diagnoses. The stroma of lymphadenoma is CD34 reactive [7]. Both basaloid and clear cells stain with cytokeratin AE1/AE3 [8]. The peripheral rim of basaloid cells also stains with BerEP4, a well-known BCC marker that is also positive in other follicular germinative cell-derived lesions such as trichoepithelioma and trichoblastoma [5, 9]. Nevertheless, lymphadenoma is distinguished from BCC based on its lack of peripheral palisading, mucin tinted retraction space, mitotic, and apoptosis figures [3]. Cytokeratin 20 (CK20) and CK17 have also been utilized to distinguish CL from BCC. The CK20+ Merkel cells often infiltrate the basaloid islands of CL but are usually absent in BCC. The BCC lesions demonstrate diffuse CK17 staining, whereas the tumoral islands in CL show a peripheral rim or patchy staining pattern [10].

Other histopathological differential diagnoses include clear cell syringoma, lymphoepithelioma-like carcinoma of the skin (LELC), and dermal thymus. Clear cell syringoma lacks the prominent lymphocytic infiltration and consists of a sclerotic stroma in which clear cells form tadpole-shaped ducts and islands. Syringoma is S-100, CEA, EMA, lysozyme, GCDFP-15, and GCDFP-24 positive [1]. The LELC is also composed of epithelial nests and lymphocytic infiltration, but it has a single linage of polygonal epithelioid cells with prominent cytological atypia that are derived from sweat glands. LELC is positive for EMA, and p63, and negative for S-100 [11]. The dermal thymus represents an ectopically located thymic tissue that is almost always associated with the branchio-oculofacial syndrome. Histologically, it consists of lymphoid tissue and Hassall's corpuscles [2].

Simple excision is currently the treatment of choice in CL. Since recurrence and metastasis have not been reported to date, the reemergence of the lesion, in this case, might be attributed to the insufficient margin of shave excision [3]. Additionally, if the tumor margins are not clinically well defined, Mohs surgery is particularly useful in such cases to ensure complete removal of the lesion. Mohs surgery might also be beneficial in cases that are located in anatomically sensitive areas where tissue preservation is critical [12].

## Data Availability

No data were used to support this study.
